# Preventing lipophilic aggregation in cosolvent molecular dynamics simulations with hydrophobic probes using Plumed Automatic Restraining Tool (PART)

**DOI:** 10.1186/s13321-024-00819-y

**Published:** 2024-02-27

**Authors:** Olivier Beyens, Hans De Winter

**Affiliations:** Laboratory of Medicinal Chemistry, Department of Pharmaceutical Sciences, University of Antwerp, Universiteitsplein 1, 2610 Wilrijk, Belgium

**Keywords:** Molecular dynamics, Cosolvent simulations, Metadynamics, Conformational sampling, Drug design

## Abstract

Cosolvent molecular dynamics (MD) simulations are molecular dynamics simulations used to identify preferable locations of small organic fragments on a protein target. Most cosolvent molecular dynamics workflows make use of only water-soluble fragments, as hydrophobic fragments would cause lipophilic aggregation. To date the two approaches that allow usage of hydrophobic cosolvent molecules are to use a low (0.2 M) concentration of hydrophobic probes, with the disadvantage of a lower sampling speed, or to use force field modifications, with the disadvantage of a difficult and inflexible setup procedure. Here we present a third alternative, that does not suffer from low sampling speed nor from cumbersome preparation procedures. We have built an easy-to-use open source command line tool PART (Plumed Automatic Restraining Tool) to generate a PLUMED file handling all intermolecular restraints to prevent lipophilic aggregation. We have compared restrained and unrestrained cosolvent MD simulations, showing that restraints are necessary to prevent lipophilic aggregation at hydrophobic probe concentrations of 0.5 M. Furthermore, we benchmarked PART generated restraints on a test set of four proteins (Factor-Xa, HIV protease, P38 MAP kinase and RNase A), showing that cosolvent MD with PART generated restraints qualitatively reproduces binding features of cocrystallised ligands.

## Introduction

Cosolvent molecular dynamics have recently gained interest as a tool for structure-based drug design using a fragment-based approach. Cosolvent molecular dynamics simulations are molecular dynamics (MD) simulations in which a user-specified amount of the water solvent molecules surrounding a protein target are replaced by so-called cosolvent molecules, where these cosolvent molecules are also termed probes. The positions of the cosolvent molecules throughout the trajectory are then analyzed a posteriori to create density maps of where the probes most often reside during the molecular dynamics simulations. A variety of probe types and mixes of probe types have been used throughout literature, such as water-benzene-isopropane, water-isopropanol or water-isopropanol-acetamide-acetate-isopropylamine [1–5]. A more comprehensive review of the different type of probe mixtures used can be found in the miniperspective written by Ghanakota and Carlson [6]. Recently, a variety of enhanced sampling techniques have also been used in combination with cosolvent molecular dynamics to further enhance performance. In SWISH, Hamiltonian replica exchange is combined with a benzene cosolvent, leading to a methodology that is promising for discovering cryptic pockets [7]. In accelerated ligand-mapping MD, accelerating molecular dynamics is combined with benzene cosolvent, leading to a methodology in which occluded binding pockets can be discovered as well [8].

An interesting application to structure-based drug design consists of building pharmacophore models from the calculated probe densities. Pharmacophore models are three-dimensional maps of chemical features thar are considered important for ligand binding [9]. By screening a library of molecules for that three-dimensional arrangement of chemical features, novel inhibitor scaffolds for protein targets can be discovered. These cosolvent-based pharmacophore models outperform more conventional docking approaches, as was shown by the SILCS-Pharm approach [10]. Another workflow that combines cosolvent molecular dynamics with pharmacophore modeling is the Pharmmaker tool [11].

As hydrophobic and aromatic groups are often included in pharmacophore screenings, it is of interest to include these hydrophobic probes in cosolvent molecular dynamics [12]. However, a major concern in simulations with hydrophobic probe molecules, such as benzene, isopropane or isobutane, is that phase separation can occur by aggregation of these hydrophobic probes. Bakan et al*.* [3] mentioned that isobutane is one the most common fragment types in approved drugs, however isobutane was not included in the probe mix due to the insolubility of isobutane and risk for aggregation. The Site Identification by Ligand Competitive Saturation (SILCS) approach by Guvench et al*.* [2, 10, 13–18] provides a working solution to this aggregation risk by including an artificial repulsion term between the hydrophobic probes. This artificial repulsion term is incorporated by adapting the corresponding non-bonded force field parameters. However, modifying force fields is a rather complex procedure, and additionally force field cut-offs need to be adapted from the standards for which the force field was parametrized [19]. Another possibility to avoid hydrophobic probe aggregation is to use simulations with lower probe concentrations (<0.2 M) [8, 20]. However, for applications where faster convergence in probe density maps is desired, the low sampling speed stemming from the low concentrations might be detrimental. Consequently, there is a need for a flexible and easy to setup methodology that generates intermolecular repulsion interactions between hydrophobic probes in molecular dynamics simulations, and in this context we developed a workflow using PLUMED-based restraints [21–23]. In this paper we present the “Plumed Automatic Restraining Tool” (PART), a Python command line tool that prepares a PLUMED input file to integrate in cosolvent molecular dynamics workflows with hydrophobic probes without aggregation issues. In this manuscript, we detail the PART methodology, show a benchmark of a restrained versus unrestrained simulation and we demonstrate that cosolvent MD simulations with restraints generated by PART may reproduce key ligand features. The PART program, together with some usage examples is freely available on GitHub at the following link: https://github.com/UAMC-Olivier/PART.

## Methods

The methodology of the tool development consists of four parts, namely an explanation of the tool code, a comparison between unrestrained and restrained simulations to show that artificial repulsion is necessary at high hydrophobic probe concentration, a benchmark to show that known ligand features are reproduced by cosolvent simulations with intermolecular repulsion terms generated by PART, and lastly a benchmark on the slowdown of the simulation by including PART restraints.

### Plumed Automatic Restraining Tool (PART)

**Fig. 1:**
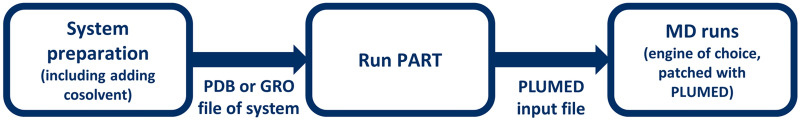
Schematic visualization of the inclusion of PART in a cosolvent molecular dynamics workflow

The Plumed Automatic Restraining Tool is a Python command line tool that generates a PLUMED input file to prevent aggregation of user-defined probe molecules using the existing “many restraints” module of PLUMED [21–23]. The general methodology consists of calculating all distances between the defined molecules, and then calculating the to-be-applied artificial repulsion using a bias function, which is user customizable if desired. The input consists of a Protein Data Bank (PDB) or GROMACS (GRO) structure file of the system with cosolvent molecules and a description of the molecule types to which the restraining potential needs to be applied. It is possible to add a restraining potential between molecules of the same type (such as benzene-benzene), or between molecules of different types (such as benzene-propane), or any combination of these. The calculation of the intermolecular distances is point-based. The user can either specify a central atom of the probe to be used as the point on which the distance calculation is based, or specify a list of atoms from which the center of mass (COM) will be computed. PART can easily be integrated in a cosolvent molecular dynamics workflow as depicted in Fig. 1.

PART will first parse the user-specified PDB or GRO file using a custom structure file parser implemented in the PART code. PART will then build all necessary atom groups and write the requested center of mass calculation to the PLUMED file. As a final step, the tool will write out all distance calculation statements, all restraint statements, and print statements of the bias values. The restraints statements make use of lower walls, of which the limit and harmonic potential terms are user-customizable if desired using optional command line flags. The default parameter values for the artificial repulsion potential $V$ are an energy constant $k$ of approximately 0.5 kcal mol^−1^ Å^−4^, a wall location $a$ of 8.0 Å, a scaling factor $s$ of 1.0 and an exponent $e$ of 4.0 with $x$ being the intermolecular distance in Å (Eq. 1):

1 $$V=k{\left(\frac{x-a}{s}\right)}^{e} \, if \, x<a , V=0 \, if \, x\ge a$$

After running PART, the PLUMED file can be further modified by the user to include additional restraints, such as protein root-mean-square deviation restraints, if desired.

### Comparison between restrained and unrestrained cosolvent MD simulations

The Factor Xa protein (PDB: 1FJS) [24] structure was prepared by removing ligands and glycerol molecules, while retaining crystal waters and ions. Side chain flips were analyzed with Reduce [25] and protonation states of the ionizable groups with PROPKA [26, 27]. Occurrence of disulfide bridges was checked manually. Molecular dynamics engine of choice was GROMACS 2021.3 [28, 29] patched with PLUMED 2.7.2 [21–23], the chosen force field was CHARMM36m (July 2021 version) [19, 30]. Consequently, the prepared PDB file of the protein was converted to GROMACS format, and the system was placed in a periodic dodecahedral box, where the box edges are at least 12 Å away from any protein element. A workflow tool involving the PART tool was used for adding the cosolvent molecules, counterions and original TIP3P [31] water molecules at ten different randomly generated starting locations. The chosen cosolvent molecules were the same as in the SILCS multiple fragment types methodology [16], namely benzene, propane, methanol, formamide, acetaldehyde, acetate and methylammonium. A PART generated PLUMED file with the intermolecular repulsion terms between benzene-benzene, propane-benzene, propane-propane and acetate-methylammonium was also generated at this stage. Force field parameters for the cosolvent molecules were generated using CGenFF (CGenFF version 4.6, CGenFF program version 2.5) [32–35]. The concentration of each cosolvent molecule type was set to 0.25 M.

After system preparation, the system was minimized in two stages: a first stage with flexible waters and without hydrogen bond constraints, followed by a second stage with rigid waters and with hydrogen bond constraints. Throughout the second minimization stage and all subsequent simulations, hydrogen bonds were constrained using the LINCS algorithm [36]. Minimization was set to steepest descent with a maximum number of 50,000 steps and the default convergence criterion. Minimization step size was 0.01 Å for the first stage and 0.1 Å for the second. The system was then initialized at a temperature of 300 K equilibrated in three stages: one in the NVT ensemble with positional restraints on the protein heavy atoms, one in the NPT ensemble, again with positional restraints, and one in the NPT ensemble without positional restraints. The force constant for positional restraints was approximately 2.4 kcal mol^−1^ Å^−2^. The NVT equilibration was carried out over 0.5 ns, while both NPT stages of the equilibration were simulated for 1 ns.

The actual production runs in the NPT ensemble were simulated for 100 ns for each of the ten different randomly generated starting coordinates, leading to a total simulation length of 1 µs. Throughout all simulations, force field cut offs of 12 Å were used, where the potential is smoothly switched to zero between 10 Å and 12 Å. Long range electrostatics were treated using Particle Mesh Ewald (PME) [37] method. Temperature coupling in all simulations was carried out using the V-rescale thermostat [38] with a time constant of 0.1 ps, with the reference temperature set to 300 K. Protein and non-protein elements were coupled separately. Pressure coupling was executed by the C-rescale barostat [39] with a time constant of 2.0 ps and with a reference pressure of 1 bar in all NPT ensemble simulations.

The above simulation protocol was applied twice to Factor Xa, once with the PART generated PLUMED restraints and once without. For the simulation with PART restraints, the PART default potential was applied for both NPT equilibrations and for the production simulations. For the NVT equilibration, a softer potential (parameters: $k\approx$ 0.02 kcal mol-1 Å^−2^, $a$ = 8.0 Å, $s$ = 1.0, $e$ = 2.0) was applied as to not introduce large forces to cosolvent molecules close to each other.

During simulation analysis, radial distribution functions (RDFs) were calculated by binning the individual intermolecular distances over the MD trajectories of the ten simulation replicas. Chosen distance calculation points were the COM of benzene, the central carbon atom of propane, the nitrogen atom of methylammonium, the carboxylic carbon atom of acetate and the carbon atom of formamide.

We also analyzed the percentage of ionic organic fragments participating in ion-ion interactions and the percentage of aggregated lipophilic molecules. To examine this, we computed the distances between the molecules of interest by calculating the intermolecular distance matrices. For each MD frame, the number of molecules participating in interactions was then calculated based on these intermolecular distance matrices. The criterium for ionic interactions was a distance below 4 Å. The criterium for a hydrophobic molecule to participate in lipophilic aggregation was modelled as one intermolecular distance to another hydrophobic molecule below 7 Å. Finally, the average fraction of molecules participating in interactions was calculated as the average of interacting molecules over all the MD frames from the ten replicas. The distance calculations were performed using PLUMED.

### Ligand feature reproduction benchmarks

We benchmarked the PLUMED restraints generated by PART on the results found in a SILCS benchmark by Raman et al*.* on four protein systems, namely Factor-Xa (PDB:1FJS) [24], HIV protease (PDB:1G2K) [40], P38 MAP kinase (PDB:1OUY) [41] and RNase A (PDB:1JVT) [42]. An identical cosolvent mixture as in the previously detailed restrained versus unrestrained MD simulations was used. Raman et al*.* [16] compared SILCS fragment affinity maps to important binding features of cocrystallised ligands to the four benchmark proteins, of which we analyzed a selection of ligands as well. Additionally, we analyzed whether our simulations can reproduce key ligand features of a set of recently published crystal structures of RNase A [43]. An overview of the analyzed ligand–protein structures can be found in Table 1. Protein preparation and simulation protocols for the three extra proteins were the same as for the previously detailed restrained Factor Xa benchmark, with one exception: for the RNase A system, an additional restraint on the Cα carbons with a force constant of approximately 0.01 kcal mol^−1^ Å^−2^ was added during the second NPT equilibration and the MD production to prevent protein unfolding. Visualization was performed using PyMol (Version 2.4.1, Schrödinger LLC) and density analyses were performed using MDAnalysis [44, 45]. To allow comparison with previous executions of this benchmark [16], we calculated the densities of five atom groups: hydrophobic (propane and benzene carbon atoms), hydrogen bond donor (formamide and methanol donor hydrogen atoms), hydrogen bond acceptor (formamide, methanol and acetaldehyde oxygen atoms), negatively charged (acetate oxygen atoms) and positively charged (methylammonium polar hydrogens). The atom densities were calculated as an average over the ten replicas per protein. These groupwise summed atom density grids were converted to grid free energies (GFEs) using the below described formula, where $R$ being the ideal gas constant, $T$ is the temperature, $n$ is the calculated density for the grid voxel under study and ${n}_{expected}$ is the expected density, calculated by dividing the total number of atoms of the atom group in the box by the average box volume throughout the simulation (Eq. 2):

**Table 1:** Overview of the analyzed ligands in the ligand feature reproduction benchmark

Protein system	PDB codes of analyzed ligands
Factor-Xa	1FJS [24], 1EZQ [46], 1MQ5 [47], 1Z6E [48]
HIV protease	1G2K [40], 1DMP [49], 1B6K [50], 1D4L [51]
P38 MAP kinase	1OUY [41], 1W84 [52], 1A9U [53], 1BL7 [53], 1DI9 [54], 1WBW [52]
RNase A	1O0H [55], 1O0O [55], 1O0M [54], 1QHC [56], 6PVV* [43], 6PVX* [43]

*Not included in the original SILCS [16] benchmark set

2 $$GFE= -RT \text{ln(}\frac{n}{{n}_{expected}}\text{)}$$

### Performance benchmarks

We have calculated a performance test of the PART restraints in which we measure the percentage of the total simulation time going to PART restraints as a function of different hardware architectures and different probe mixtures. We tested four hardware architectures, namely 16, 64 and 128 cores of an AMD EPYC 7H12 CPU and 12 cores of an AMD Epyc 7402 CPU combined with one Nvidia A100 GPU. The system setup was similar to the Factor-Xa benchmark, with as only difference the probe concentrations. We tested seven probe mixtures in total. The first mixture was set up as an exact copy of the Factor-Xa benchmark. The second and third system used only propane as cosolvent, at concentrations of 0.25 M and 0.5 M respectively. The fourth and fifth system used only benzene as cosolvent, also at concentrations of 0.25 M and 0.5 M. Comparing the propane and benzene only mixtures allows to study the influence of single atom versus COM-based distance calculations, as for propane a single atom was used and for benzene the COM of all benzene atoms. To study the influence of the number of atoms in a COM simulation, we made a sixth and seventh experiment in which we use the previously described benzene only mixtures, but with the COM calculation based on the benzene carbon atoms only. We used short MD simulations of 100 ps for the scaling tests.

## Results and discussion

### Comparison between restrained and unrestrained simulations

**Fig. 2:**
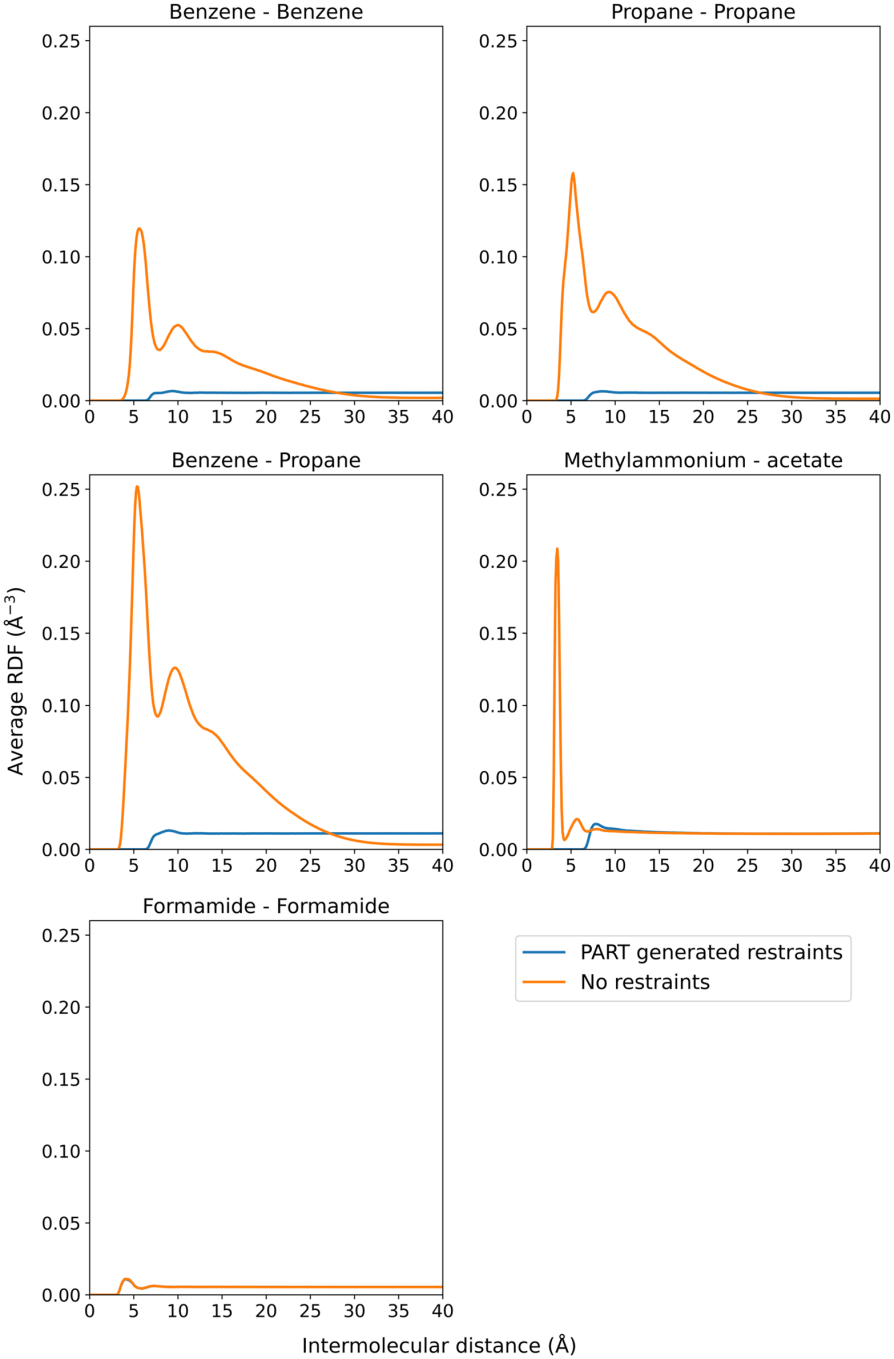
Radial distribution functions for a selection of probe pairs for the Factor-Xa simulations with (blue curves) and without (orange curves) PART restraints. Radial distribution functions were calculated as an average over the ten MD replicas of the simulations with and without PART restraints. As a reference, the RDF for formamide is also shown, a molecule for which aggregation does not take place, hence the simulations with and without PART generated restraints have the same, overlapping RDF curve

**Fig. 3:**
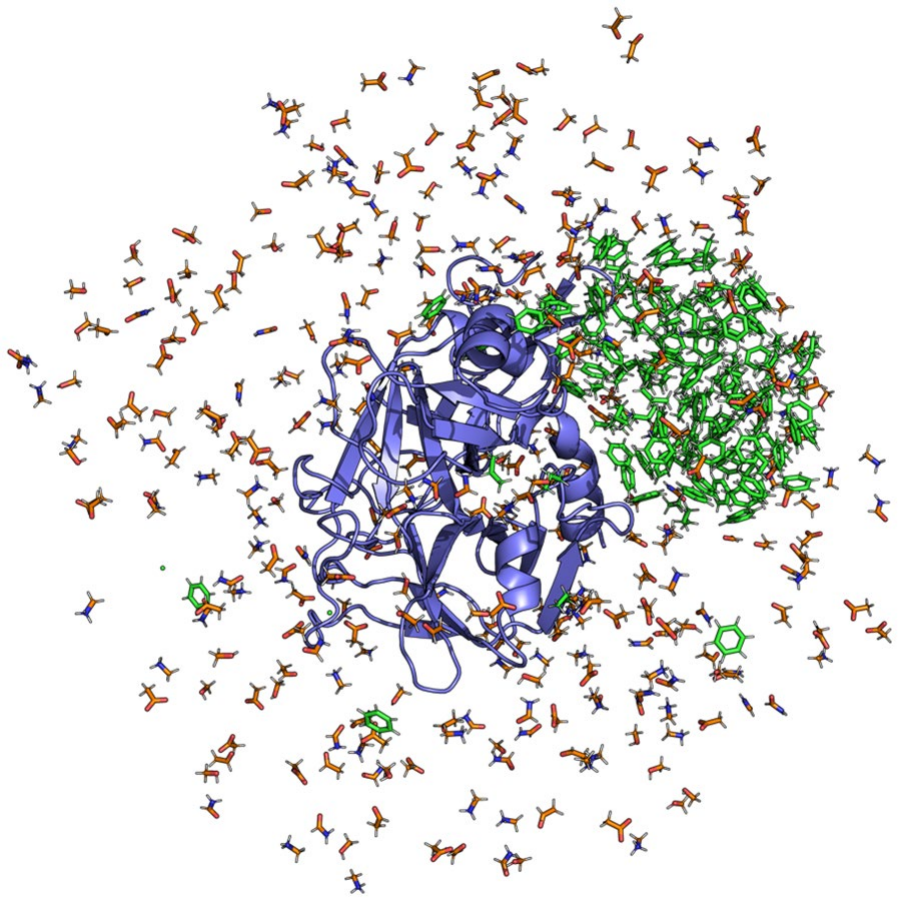
Illustration of lipophilic aggregation between hydrophobic probes (green) during the Factor-Xa protein (purple) simulation without PART restraints. Water soluble probes are shown in orange

Figure 2 shows the radial distribution functions (RDFs) between a selection of cosolvent molecule types. These RDFs were calculated for the Factor-Xa mixture without PART generated restraints and for the Factor-Xa mixture with PART generated restraints, as described earlier. If aggregation takes place, RDF curves show peaks at low intermolecular distances and have less occurrences of higher intermolecular distances. Following these criteria, aggregation is clearly taking place between benzene-benzene, propane-benzene, and propane-propane pairs. Visual inspection of the trajectory also confirms this lipophilic aggregation (Fig. 3). This analysis indicates that intermolecular distance restraints between hydrophobic molecule types are necessary at higher concentrations. From the intermolecular distance matrices and by counting the number of interactions, the average fraction of intermolecular interactions between the fragments could be calculated (see Methods section). The average fraction of hydrophobic molecules participating in lipophilic aggregation was 88%, composed of 90% of propane interacting with other hydrophobic molecules, and 86% of benzenes interacting with other hydrophobic molecules. As a reference, Fig. 2 also shows the RDF for formamide, a molecule for which aggregation does not take place as it is water soluble and not formally charged.

When analyzing the ionic interactions between acetate (negative formal charge) and methylammonium (positive formal charge), a simple conclusion is less obvious. Due to the nature of these interactions, the ionic fragments form pairs rather than large aggregates. Pair formation will result in a peak at low intermolecular distances in an RDF curve, but the RDF values at longer intermolecular distances are less impacted since the ionic pairs are still randomly distributed in the simulation box and do not aggregate. The average fraction of ionic fragments making ion-ion interactions is 26.4%. As this is not as detrimental as compared to lipophilic aggregation, it is up to the user to decide whether it is worthwhile to include restraints between oppositely charged fragment types.

### Ligand feature reproduction benchmarks

**Fig. 4:**
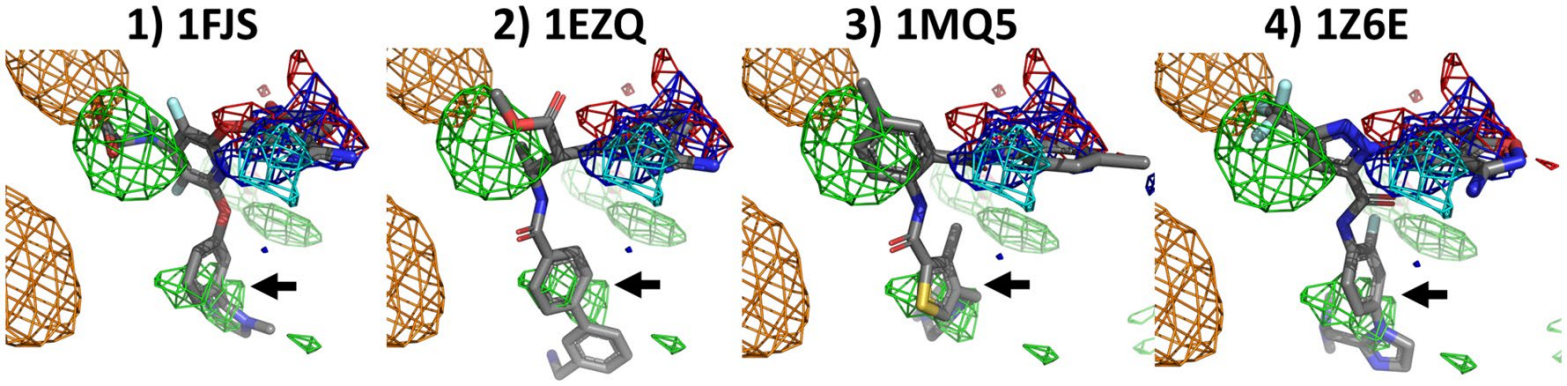
Overlap between Factor-Xa ligands and the calculated densities from a Factor-Xa cosolvent MD simulation with PART generated restraints. All grid points with GFE values of less or equal to −1.5 kcal/mol are shown as a mesh (green: hydrophobic, dark blue: donor, red: acceptor, orange: negatively charged, cyan: positively charged). The black arrow highlights a hydrophobic density mesh that overlaps with a ring feature in all four ligands

Figure 4 shows the fragment densities in Factor-Xa, as calculated from the cosolvent MD trajectory, and overlaid with several ligand structures. Positively charged fragment densities overlap nicely with ligand benzamidine groups, while negatively charged fragment density overlaps with the 1FJS ligand carbonic acid group. The trifluoromethyl group of the 1Z6E ligand is reproduced by the hydrophobic fragments, while hydrophobic rings in all four ligands (see black arrow) are also located in hydrophobic density sites.

**Fig. 5:**
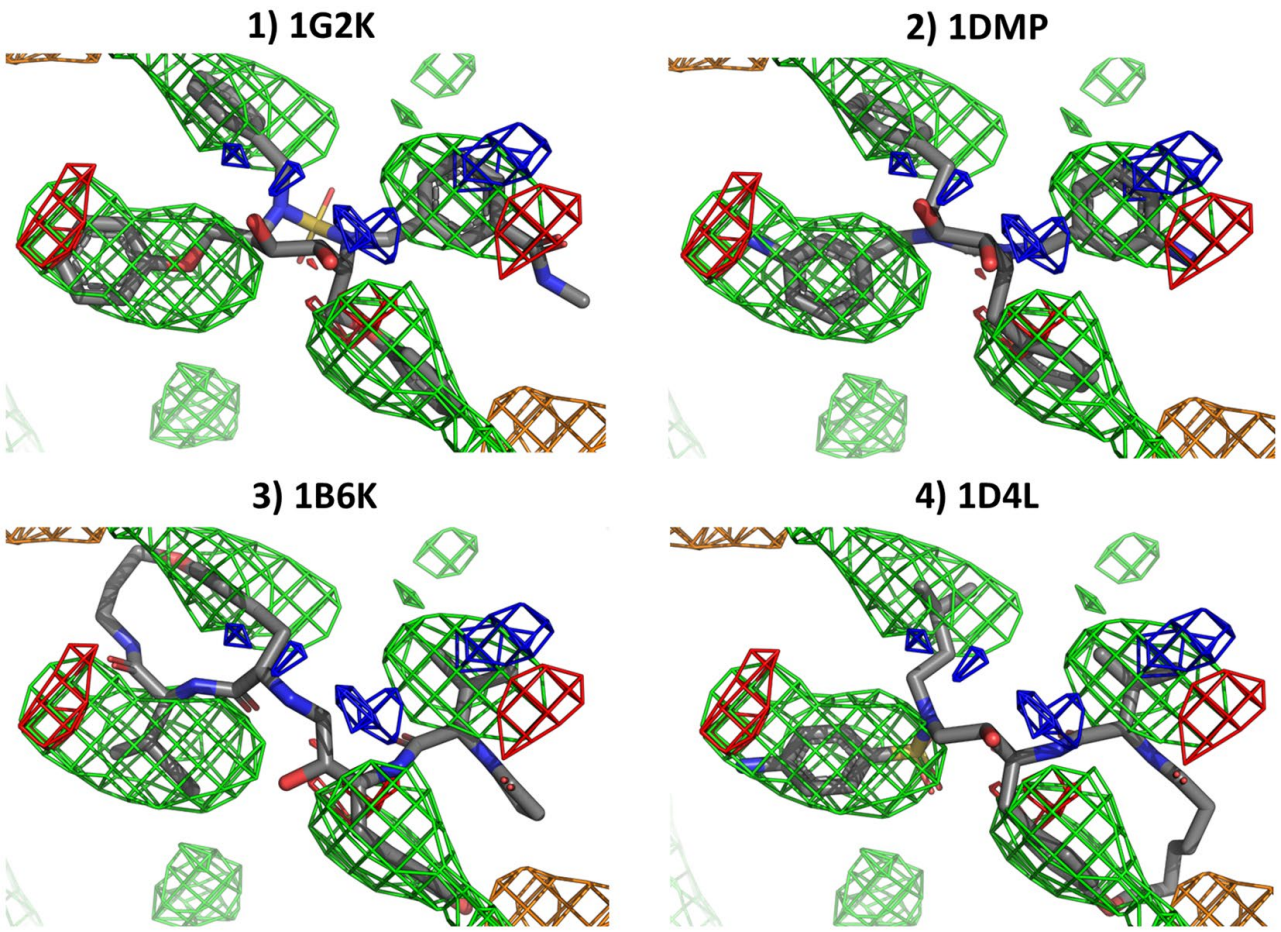
Overlap between HIV protease ligands and the calculated densities from a HIV protease cosolvent MD simulation with PART generated restraints. All grid points with GFE values ≤ −1.5 kcal/mol are shown as a mesh (green: hydrophobic, dark blue: donor, red: acceptor, orange: negatively charged, cyan: positively charged)

HIV protease fragment densities (Fig. 5) also reproduce some key hydrophobic ligand features, including all four phenyl groups from the 1G2K and 1DMP ligands and the lipophilic macrocycle of the last two ligands. Hydrogen bond donor and hydrogen bond acceptor features are in general divided between spurious and non-spurious (such as the amide oxygen in the 1G2K ligand) sites. Of note is that none of the fragments producing the donor and acceptor maps are directly influenced by PART generated restraints.

**Fig. 6:**
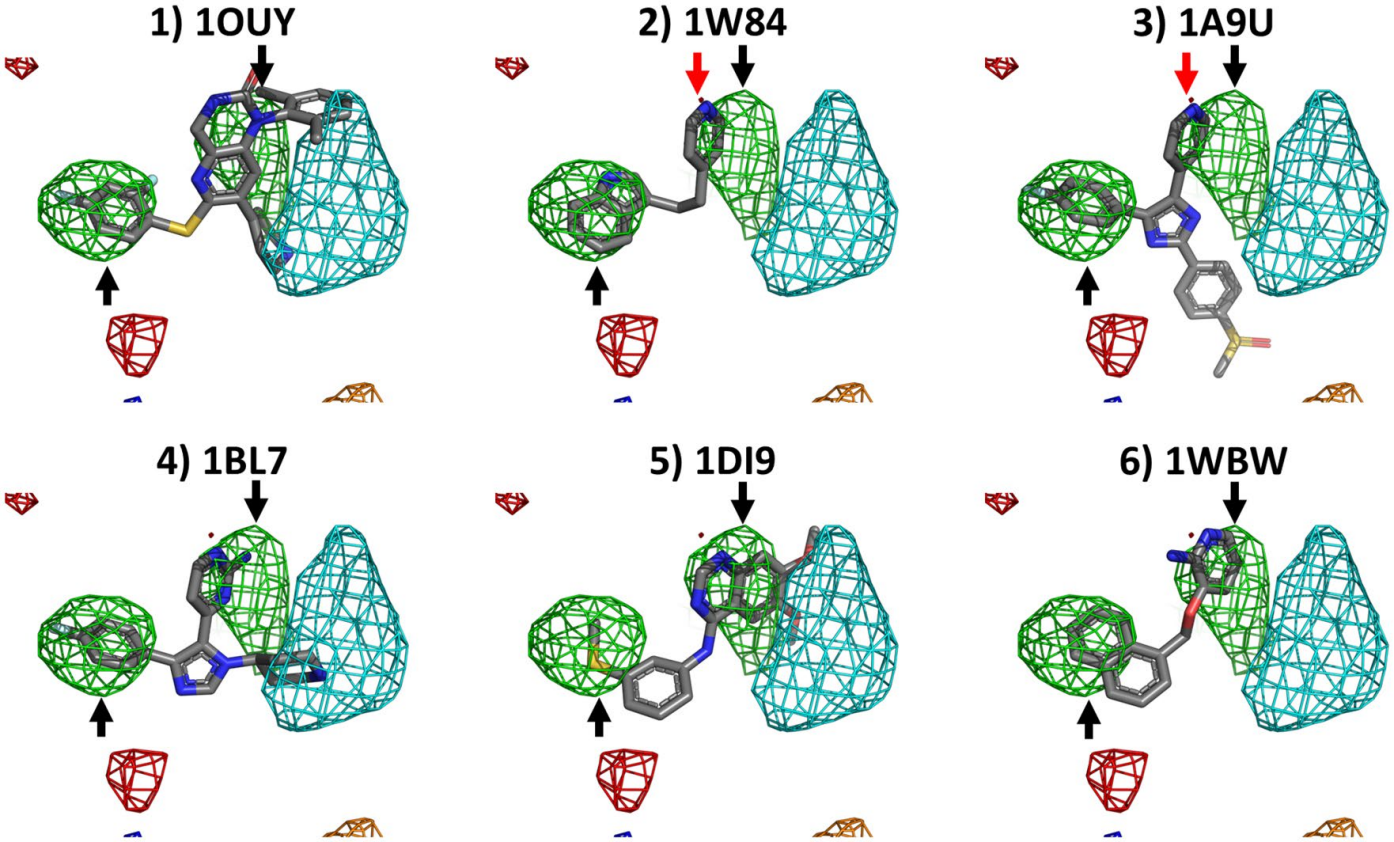
Overlap between P38 MAP kinase ligands and the calculated densities from a HIV protease cosolvent MD simulation with PART generated restraints. All grid points with GFE values ≤ −1.5 kcal/mol are shown as a mesh (green: hydrophobic, dark blue: donor, red: acceptor, orange: negatively charged, cyan: positively charged). The black arrow highlights a hydrophobic density mesh that overlaps with the ring systems in the ligands. The red arrow highlights an acceptor density that overlaps with the pyridine nitrogen atom of 1W84 and 1A9U

In the P38 MAP kinase benchmark, shown in Fig. 6, the hydrophobic map type corresponds to the ring systems in the ligands, as indicated by the two black arrows. The secondary amine of the 1OUY and 1BL7 ligand is also positioned inside the positive fragment density. Interestingly, the pyridine nitrogen atom of 1W84 and 1A9U also overlaps with an acceptor density, as indicated by the red arrow, although again acceptor densities are not influenced by PART.

**Fig. 7:**
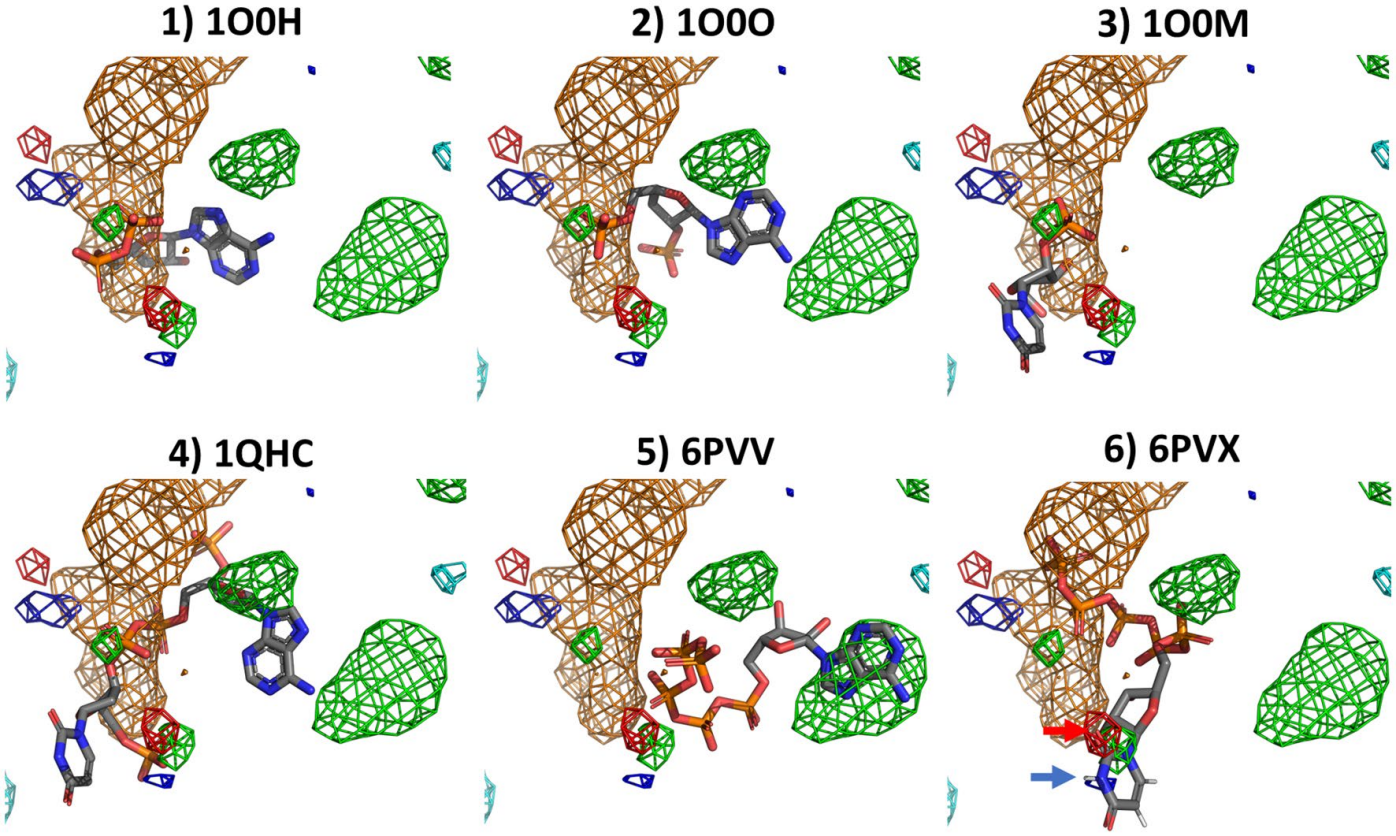
Overlap between RNase A ligands and the calculated densities from a HIV protease cosolvent MD simulation with PART generated restraints. All grid points with GFE values ≤ −1.5 kcal/mol shown as a mesh (green: hydrophobic, dark blue: donor, red: acceptor, orange: negatively charged, cyan: positively charged). The red arrow highlights a hydrogen bond acceptor density that overlaps with an acceptor feature of the 6PVX ligand. The blue arrow highlights a donor density that overlaps with a donor feature of the 6PVX ligand

Negatively charged phosphate groups are in general close to, or inside of, negatively charged fragment densities in the RNase A benchmark, as illustrated in Fig. 7. The aromatic system of the 6PVV ligand overlaps with calculated hydrophobic/aromatic fragment densities. Additionally, donor and acceptor densities overlap with donor and acceptor locations in the 6PVX ligand, as indicated by the blue and red arrows.

In general, we conclude from the above results that important ligand features can be qualitatively reproduced by cosolvent MD simulations with PART generated restraints. If using a high concentration of hydrophobic probes, intermolecular restraints will ensure that fragments densities accumulate in the appropriate pockets at an expected sampling speed. These results are in line with benchmarks done using other methodologies such as SILCS [16].

### Performance benchmarks

The results from the performance benchmarks are shown in Table 2. Clearly the PLUMED part of the calculation does not scale as well over increasing cores as the GROMACS part of the calculation. When making use of a GPU, the GROMACS part of the calculation can become faster than PLUMED part of the calculation, severely impacting total simulation speed. Consequently, we advise users of PART to make use of CPU partitions, while using one full CPU per simulation and run multiple replicas in parallel. We note that if future PLUMED code is better optimized for the calculations used in PART, then these scaling test results might change.

**Table 2:** Overview of the PART performance benchmark showing the percentage of the total simulation time going to PART restraints as a function of different hardware architectures and different probe mixtures

	16 CPU cores	64 CPU cores	128 CPU cores	1 A100 GPU
Mix 1 PLUMED time (%)	13	22	36	71
Mix 2 PLUMED time (%)	3	6	22	43
Mix 3 PLUMED time (%)	5	12	28	50
Mix 4 PLUMED time (%)	8	12	28	62
Mix 5 PLUMED time (%)	12	21	36	66
Mix 6 PLUMED time (%)	4	10	26	50
Mix 7 PLUMED time (%)	10	18	34	68

Mix 1 is a copy of the Factor-Xa benchmark where all benzene atoms where used to calculate the COM. Mix 2 is the Factor-Xa system where the only cosolvent is propane at a concentration of 0.25 M and Mix 3 is a copy of Mix 2 but with a probe concentration of 0.5 M. Mix 4 is the Factor-Xa system where the only cosolvent is benzene at a concentration of 0.25 M. Mix 5 is a copy of Mix 4 but with a probe concentration of 0.5 M. In Mix 4 and Mix 5, the COM of benzenes was calculated using all benzene atoms. Mix 6 and Mix 7 are a copy of Mix 4 and Mix 5 respectively, but now calculating the COM using only the benzene carbon atoms

## Conclusion

We have shown that PART is a new easy-to-use alternative for current methodologies for cosolvent MD simulations involving hydrophobic cosolvent molecules. The main advantages of PART are short setup times (especially compared to force field modifications where these modifications need to be validated), a restraint potential that can be easily modified, and that the recommended force field cutoffs can be used. Regarding the last point, in competing technologies such as SILCS, force field cut-offs are modified to make sure that the restraint potential introduced by force field modifications have the correct shape. Such force field cut-off changes are not required with PART-generated restraint potentials.

The main disadvantage of PART is that an extra PLUMED overhead time is introduced to the calculation. It is up to the user to decide which arguments are more important when choosing a methodology.

Additionally, we have demonstrated that restrained simulations increase the effective concentration of hydrophobic probes by preventing aggregation. Cosolvent MD simulations involving PART-generated restraints on four benchmarked proteins also reproduce known ligand features qualitatively.

## Data Availability

Project name: PART. Project home page: https://github.com/UAMC-Olivier/PART. Archived version: N/A. Operating system(s): Platform independent. Programming language(s): Python. Other requirements: MD engine of choice patched with PLUMED, numpy module for Python. License: MIT license.
